# Tetra­potassium hepta­cyanido­molybdate(III) dihydrate

**DOI:** 10.1107/S1600536809039336

**Published:** 2009-10-17

**Authors:** Koji Nakabayashi, Keisuke Tomono, Yoshihide Tsunobuchi, Wataru Kosaka, Shin-ichi Ohkoshi

**Affiliations:** a Department of Chemistry, School of Science, University of Tokyo, 7-3-1 Hongo, Bunkyo-Ku, Tokyo 113-0033, Japan

## Abstract

The asymmetric unit of the title compound, K^I^_4_[Mo^III^(CN)_7_]·2H_2_O, consists of one [Mo(CN)_7_]^4−^ anion, four K^+^ cations, and two water mol­ecules. The [Mo^III^(CN)_7_]^4−^ anion has a seven-coordinated capped-trigonal-prismatic coordination geometry. The site-occupancy factors of the disordered water mol­ecules were set at 0.90, 0.60 and 0.50. The H-atom positions could not be determined for two of the water mol­ecules. The H atoms of the water with a site-occupancy factor of 0.90 were refined using O—H and H⋯H distance restraints.

## Related literature

For the synthesis and spectroscopic information for the title compound, see: Young (1932 ▶); Rossman *et al.* (1973 ▶). For octa­cyanido­metalate-based materials with photomagnetic and magnetic properties, see: Arimoto *et al.* (2003 ▶); Catala *et al.* (2005 ▶); Ohkoshi *et al.* (2007 ▶, 2008 ▶). For the related hepta­cyanido molybdate(III) crystal structure with *D_5h_* and *C_2v_* symmetry, see: Hursthouse *et al.* (1980 ▶)); Larionova *et al.* (2004 ▶); For a hepta­cyanido molybdate(II) crystal structure with *C_s_* symmetry, see: Drew *et al.* (1977 ▶).
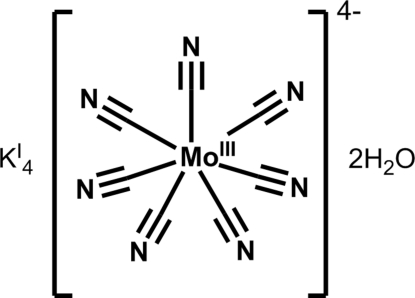


## Experimental

### 

#### Crystal data

K_4_[Mo(CN)_7_]·2H_2_O
*M* *_r_* = 468.29
Triclinic, 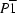

*a* = 8.8813 (5) Å
*b* = 9.2896 (4) Å
*c* = 9.7221 (4) Å
α = 86.6480 (13)°
β = 82.2150 (19)°
γ = 71.2570 (17)°
*V* = 752.48 (6) Å^3^
*Z* = 2
Mo *K*α radiation
μ = 1.99 mm^−1^
*T* = 90 K
0.10 × 0.05 × 0.02 mm

#### Data collection

Rigaku R-AXIS RAPID diffractometer
Absorption correction: multi-scan (*ABSCOR*; Higashi, 1999 ▶) *T*_min_ = 0.826, *T*_max_ = 0.961
7367 measured reflections
3421 independent reflections
2893 reflections with *I* > 2σ(*I*)
*R*_int_ = 0.025

#### Refinement

*R*[*F* ^2^ > 2σ(*F* ^2^)] = 0.054
*wR*(*F* ^2^) = 0.153
*S* = 1.25
3421 reflections
210 parameters
3 restraints
H-atom parameters not defined
Δρ_max_ = 2.85 e Å^−3^
Δρ_min_ = −1.08 e Å^−3^

### 

Data collection: *PROCESS-AUTO* (Rigaku, 1998 ▶); cell refinement: *PROCESS-AUTO*; data reduction: *CrystalStructure* (Rigaku, 2007 ▶); program(s) used to solve structure: *SHELXS97* (Sheldrick, 2008 ▶); program(s) used to refine structure: *SHELXL97* (Sheldrick, 2008 ▶); molecular graphics: *CrystalStructure* (Rigaku, 2007 ▶); software used to prepare material for publication: *ORTEP-3* (Farrugia, 1997 ▶) and *PyMOLWin* (DeLano, 2007 ▶).

## Supplementary Material

Crystal structure: contains datablocks I, global. DOI: 10.1107/S1600536809039336/si2189sup1.cif

Structure factors: contains datablocks I. DOI: 10.1107/S1600536809039336/si2189Isup2.hkl

Additional supplementary materials:  crystallographic information; 3D view; checkCIF report

## Figures and Tables

**Fig. 1.**
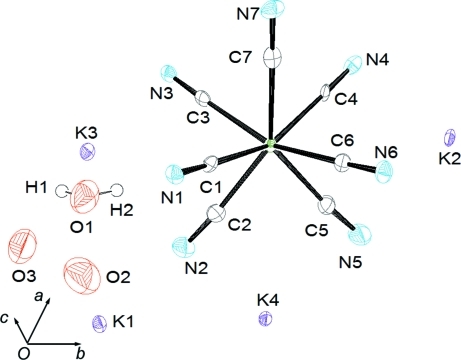
Thermal ellipsoid plots (50% probability level) of independent atoms of KI4[MoIII(CN)7]2H2O. Positions of hydrogen atoms of the disordered water molecules could not be determined.

## supplementary crystallographic information

### Comment

In the field of molecule-based magnets, cyano-bridged metal assemblies have been much studied to demonstrate novel functionalities. Octacyanometalate-based materials show interesting functionalities such as photomagnetism (Arimoto *et al.*, 2003; Catala *et al.*, 2005; Ohkoshi *et al.*, 2008) and chemically sensitive magnetism (Ohkoshi *et al.*, 2007). As a versatile class of building blocks, a heptacyanomolybdate ion [Mo^III^(CN)_7_]^4-^ is also attractive because this metal complex can adopt various spatial configurations, depending on the chemical environment, pentagonal bipyramid (*D*_5 h_) and capped trigonal prism (*C*_2v_) (Rossman *et al.*, 1973; Larionova *et al.*, 2004). Although the coordination geometry of pentagonal bipyramid (*D*_5 h_) has been observed with the mixed salt Na^I^K^I^_3_[Mo(CN)_7_]2H_2_O, that of capped trigonal prism (*C*_2 v_) has not been reported (Hursthouse *et al.*, 1980, Drew *et al.*, 1977). In this paper, we report the crystal structure of K^I^_4_[Mo^III^(CN)_7_]2H_2_O with capped trigonal prism (*C*_2v_) symmetry, which has the asymmetric unit of one [Mo^III^(CN)_7_]^4-^ anion, four K^+^ cations, and two water molecules distributed over three sites with site occupation factors (s.o.f's) of 0.90, 0.60 and 0.50. (Fig. 1). The synthesis and the spectral information of the title compound as been reported by Young (1932) and Rossman *et al.*, (1973).

### Experimental

The title compound was prepared by reacting (NH_4_)_2_[MoCl_5_(H_2_O)] (1.81 g) with KCN (4 g) in H_2_O (23 ml) at room temperature. The prepared compound was a green plate-type crystal. Elemental analyses: calcd for K^I^_4_[Mo^III^(CN)_7_]2.75H_2_O, Calculated: Mo, 19.82%; C, 17.37%; H, 1.15%; N, 20.26%. Found: Mo, 19.44%; C, 17.65%; H, 1.13%; N, 20.01%. In the Infrared (IR) spectra, cyano stretching peaks were observed at 2114 and 2069 cm^-1^.

### Refinement

The H atoms of the solvent water molecules, O2 and O3, could not be located. The maximum and minimum residual electron density peaks were located 0.18 and 0.91 Å from the K3 atom and the Mo1 atom, respectively.

### Crystal data

**Table:**

K_4_[Mo(CN)_7_]·2H_2_O	*Z* = 2
*M**_r_* = 468.29	*F*(000) = 454
Triclinic, *P*1	*D*_x_ = 2.067 Mg m^−^^3^
*a* = 8.8813 (5) Å	Mo *K*α radiation, λ = 0.71075 Å
*b* = 9.2896 (4) Å	Cell parameters from 6568 reflections
*c* = 9.7221 (4) Å	θ = 3.0–27.5°
α = 86.6480 (13)°	µ = 1.99 mm^−^^1^
β = 82.2150 (19)°	*T* = 90 K
γ = 71.2570 (17)°	Platelet, green
*V* = 752.48 (6) Å^3^	0.10 × 0.05 × 0.02 mm

### Data collection

**Table:**

Rigaku R-AXIS RAPID diffractometer	3421 independent reflections
Radiation source: fine-focus sealed tube	2893 reflections with *I* > 2σ(*I*)
graphite	*R*_int_ = 0.025
ω scans	θ_max_ = 27.5°, θ_min_ = 3.0°
Absorption correction: multi-scan (*ABSCOR*; Higashi, 1999)	*h* = −11→11
*T*_min_ = 0.826, *T*_max_ = 0.961	*k* = −12→11
7367 measured reflections	*l* = −12→11

### Refinement

**Table:**

Refinement on *F*^2^	Primary atom site location: structure-invariant direct methods
Least-squares matrix: full	Secondary atom site location: difference Fourier map
*R*[*F*^2^ > 2σ(*F*^2^)] = 0.054	H-atom parameters not defined
*wR*(*F*^2^) = 0.153	*w* = 1/[σ^2^(*F*_o_^2^) + (0.0908*P*)^2^] where *P* = (*F*_o_^2^ + 2*F*_c_^2^)/3
*S* = 1.25	(Δ/σ)_max_ < 0.001
3421 reflections	Δρ_max_ = 2.85 e Å^−^^3^
210 parameters	Δρ_min_ = −1.08 e Å^−^^3^
3 restraints	

### Special details

**Table:**

Geometry. All e.s.d.'s (except the e.s.d. in the dihedral angle between two l.s. planes) are estimated using the full covariance matrix. The cell e.s.d.'s are taken into account individually in the estimation of e.s.d.'s in distances, angles and torsion angles; correlations between e.s.d.'s in cell parameters are only used when they are defined by crystal symmetry. An approximate (isotropic) treatment of cell e.s.d.'s is used for estimating e.s.d.'s involving l.s. planes.
Refinement. Refinement of *F*^2^ against ALL reflections. The weighted *R*-factor *wR* and goodness of fit *S* are based on *F*^2^, conventional *R*-factors *R* are based on *F*, with *F* set to zero for negative *F*^2^. The threshold expression of *F*^2^ > σ(*F*^2^) is used only for calculating *R*-factors(gt) *etc*. and is not relevant to the choice of reflections for refinement. *R*-factors based on *F*^2^ are statistically about twice as large as those based on *F*, and *R*- factors based on ALL data will be even larger. Some weak peaks appeared around all O atoms (O1, O2, and O3). Although we could assign the H atoms of O1 with restraints of *DFIX* (O—H 0.85 0.02 Å and H···H distances about 1.38 0.02 Å), the H atoms of O2 and O3 could not be placed using restraints of *DFIX*.

### Fractional atomic coordinates and isotropic or equivalent isotropic displacement parameters (Å^2^)

**Table:**

	*x*	*y*	*z*	*U*_iso_*/*U*_eq_	Occ. (<1)
Mo1	−0.28013 (4)	0.18814 (3)	0.22632 (3)	0.00989 (16)	
K4	0.28925 (10)	0.01355 (9)	0.17415 (9)	0.0124 (2)	
K1	0.27995 (11)	−0.37242 (10)	0.36699 (9)	0.0146 (2)	
K2	−0.28226 (10)	0.58985 (9)	0.03172 (9)	0.0129 (2)	
K3	−0.27856 (10)	−0.21632 (9)	0.42126 (9)	0.0133 (2)	
N3	−0.3585 (4)	−0.1085 (4)	0.1198 (4)	0.0150 (7)	
N4	−0.3673 (5)	0.3229 (4)	−0.0868 (4)	0.0182 (8)	
N1	−0.3641 (5)	0.0960 (4)	0.5568 (4)	0.0157 (7)	
N2	0.0322 (5)	−0.0987 (5)	0.2950 (5)	0.0298 (10)	
N5	0.0203 (5)	0.3068 (5)	0.0908 (5)	0.0336 (11)	
C6	−0.3280 (5)	0.4105 (4)	0.3111 (4)	0.0144 (8)	
C4	−0.3348 (5)	0.2756 (4)	0.0224 (4)	0.0127 (7)	
C5	−0.0799 (5)	0.2568 (5)	0.1380 (5)	0.0219 (9)	
C1	−0.3329 (5)	0.1282 (4)	0.4402 (4)	0.0125 (7)	
C3	−0.3286 (5)	−0.0076 (4)	0.1562 (4)	0.0136 (8)	
C2	−0.0739 (5)	0.0069 (5)	0.2682 (5)	0.0206 (9)	
N6	−0.3562 (5)	0.5301 (4)	0.3555 (4)	0.0174 (8)	
C7	−0.5434 (6)	0.2691 (4)	0.2568 (4)	0.0166 (8)	
N7	−0.6817 (5)	0.3103 (4)	0.2748 (4)	0.0209 (8)	
O1	−0.0067 (7)	−0.3954 (6)	0.2344 (7)	0.0609 (15)	0.90
O2	−0.0141 (11)	−0.2434 (11)	0.5544 (12)	0.075 (3)	0.60
O3	−0.0025 (13)	−0.4462 (11)	0.4867 (14)	0.070 (3)	0.50
H1	0.019 (9)	−0.488 (3)	0.199 (6)	0.05 (2)*	0.90
H2	0.007 (9)	−0.343 (6)	0.157 (4)	0.05 (2)*	0.90

### Atomic displacement parameters (Å^2^)

**Table:**

	*U*^11^	*U*^22^	*U*^33^	*U*^12^	*U*^13^	*U*^23^
Mo1	0.0125 (2)	0.0083 (2)	0.0092 (2)	−0.00373 (15)	−0.00109 (14)	−0.00049 (14)
K4	0.0150 (5)	0.0119 (5)	0.0117 (5)	−0.0060 (4)	−0.0028 (4)	0.0013 (4)
K1	0.0201 (5)	0.0125 (4)	0.0116 (5)	−0.0058 (4)	−0.0027 (4)	0.0011 (3)
K2	0.0167 (5)	0.0075 (4)	0.0163 (5)	−0.0065 (4)	−0.0022 (4)	0.0014 (4)
K3	0.0172 (5)	0.0080 (4)	0.0156 (5)	−0.0044 (4)	−0.0034 (4)	−0.0020 (4)
N3	0.0166 (18)	0.0182 (17)	0.0096 (16)	−0.0053 (14)	−0.0019 (13)	0.0032 (13)
N4	0.0192 (19)	0.0188 (18)	0.0187 (19)	−0.0099 (15)	0.0020 (15)	−0.0051 (14)
N1	0.0215 (19)	0.0164 (17)	0.0104 (17)	−0.0066 (14)	−0.0051 (14)	0.0010 (13)
N2	0.020 (2)	0.030 (2)	0.037 (3)	−0.0057 (17)	−0.0057 (18)	0.006 (2)
N5	0.018 (2)	0.036 (3)	0.045 (3)	−0.0082 (19)	−0.004 (2)	0.012 (2)
C6	0.018 (2)	0.0144 (18)	0.0123 (19)	−0.0064 (15)	−0.0022 (15)	−0.0001 (14)
C4	0.019 (2)	0.0074 (16)	0.0137 (19)	−0.0076 (14)	−0.0029 (15)	0.0011 (14)
C5	0.016 (2)	0.016 (2)	0.033 (3)	−0.0037 (16)	−0.0025 (18)	0.0030 (17)
C1	0.0159 (19)	0.0104 (17)	0.0136 (19)	−0.0065 (14)	−0.0026 (15)	−0.0040 (14)
C3	0.0150 (19)	0.0171 (19)	0.0077 (17)	−0.0042 (15)	−0.0020 (14)	0.0036 (14)
C2	0.018 (2)	0.023 (2)	0.023 (2)	−0.0085 (17)	−0.0036 (17)	0.0039 (17)
N6	0.024 (2)	0.0174 (18)	0.0123 (18)	−0.0094 (15)	−0.0003 (14)	0.0027 (13)
C7	0.027 (2)	0.0129 (18)	0.0099 (18)	−0.0064 (16)	−0.0014 (16)	−0.0054 (14)
N7	0.019 (2)	0.0197 (19)	0.026 (2)	−0.0070 (15)	−0.0041 (16)	−0.0097 (16)
O1	0.056 (4)	0.035 (3)	0.090 (4)	−0.013 (3)	−0.004 (3)	−0.006 (3)
O2	0.058 (6)	0.070 (6)	0.088 (7)	−0.008 (5)	−0.014 (5)	0.015 (5)
O3	0.052 (6)	0.054 (6)	0.105 (9)	−0.024 (6)	−0.009 (6)	0.022 (6)

### Geometric parameters (Å, °)

**Table:**

Mo1—C2	2.120 (4)	K2—N4^vii^	2.934 (4)
Mo1—C5	2.135 (4)	K2—N7^vii^	3.096 (4)
Mo1—C1	2.150 (4)	K2—C4	3.109 (4)
Mo1—C4	2.156 (4)	K2—N4	3.134 (4)
Mo1—C6	2.160 (4)	K2—C6	3.150 (4)
Mo1—C3	2.164 (4)	K2—N6	3.181 (4)
Mo1—C7	2.198 (5)	K2—N5	3.188 (4)
K4—N1^i^	2.836 (4)	K2—C5	3.229 (4)
K4—N2	2.888 (5)	K2—C4^vii^	3.329 (4)
K4—N3^ii^	2.948 (4)	K2—C7^vii^	3.380 (4)
K4—N3^iii^	2.983 (4)	K3—O2	2.767 (10)
K4—N7^ii^	3.076 (4)	K3—N6^viii^	2.788 (4)
K4—N4^iii^	3.119 (4)	K3—O3	2.806 (11)
K4—N5	3.141 (4)	K3—O1	2.912 (6)
K4—C3^iii^	3.185 (4)	K3—N1^ix^	2.989 (4)
K4—C2	3.250 (4)	K3—N7^ix^	3.039 (4)
K4—C4^iii^	3.274 (4)	K3—N1	3.081 (4)
K4—C3^ii^	3.317 (4)	K3—C1	3.093 (4)
K4—C7^ii^	3.362 (4)	K3—C3	3.125 (4)
K1—N4^iii^	2.783 (4)	K3—N3	3.140 (4)
K1—O3	2.888 (10)	K3—C2	3.339 (4)
K1—N2	2.900 (4)	K3—N2	3.344 (5)
K1—O2	2.921 (10)	N3—C3	1.140 (6)
K1—N7^iv^	3.031 (4)	N4—C4	1.161 (6)
K1—N6^i^	3.046 (4)	N1—C1	1.174 (5)
K1—N1^i^	3.051 (4)	N2—C2	1.167 (6)
K1—N6^iv^	3.051 (4)	N5—C5	1.161 (6)
K1—O1	3.075 (6)	C6—N6	1.154 (5)
K1—C6^i^	3.200 (4)	C7—N7	1.154 (6)
K1—C1^i^	3.210 (4)	O1—K2^viii^	3.383 (6)
K1—C7^iv^	3.362 (4)	O1—H1	0.89 (2)
K2—N3^v^	2.819 (4)	O1—H2	0.89 (2)
K2—N5^vi^	2.882 (5)	O3—O3^x^	1.005 (18)
			
C2—Mo1—C5	74.20 (16)	C5—K2—C4^vii^	129.62 (10)
C2—Mo1—C1	75.73 (16)	N3^v^—K2—C7^vii^	88.06 (10)
C5—Mo1—C1	126.45 (17)	N5^vi^—K2—C7^vii^	86.38 (12)
C2—Mo1—C4	125.11 (16)	N4^vii^—K2—C7^vii^	68.44 (11)
C5—Mo1—C4	75.08 (17)	N7^vii^—K2—C7^vii^	19.92 (11)
C1—Mo1—C4	155.91 (15)	C4—K2—C7^vii^	95.04 (10)
C2—Mo1—C6	120.52 (17)	N4—K2—C7^vii^	74.03 (10)
C5—Mo1—C6	75.93 (16)	C6—K2—C7^vii^	142.50 (11)
C1—Mo1—C6	82.82 (14)	N6—K2—C7^vii^	143.29 (11)
C4—Mo1—C6	93.98 (14)	N5—K2—C7^vii^	133.68 (11)
C2—Mo1—C3	77.55 (16)	C5—K2—C7^vii^	135.26 (11)
C5—Mo1—C3	122.98 (16)	C4^vii^—K2—C7^vii^	48.32 (10)
C1—Mo1—C3	91.60 (14)	O2—K3—N6^viii^	121.8 (2)
C4—Mo1—C3	82.69 (14)	O2—K3—O3	42.0 (3)
C6—Mo1—C3	158.53 (16)	N6^viii^—K3—O3	80.6 (2)
C2—Mo1—C7	144.39 (15)	O2—K3—O1	75.4 (3)
C5—Mo1—C7	141.38 (15)	N6^viii^—K3—O1	74.07 (13)
C1—Mo1—C7	77.75 (15)	O3—K3—O1	51.2 (3)
C4—Mo1—C7	78.21 (15)	O2—K3—N1^ix^	144.9 (2)
C6—Mo1—C7	78.68 (15)	N6^viii^—K3—N1^ix^	77.83 (11)
C3—Mo1—C7	79.88 (14)	O3—K3—N1^ix^	148.6 (2)
C2—Mo1—K2	125.94 (11)	O1—K3—N1^ix^	139.68 (15)
C5—Mo1—K2	51.79 (11)	O2—K3—N7^ix^	66.5 (2)
C1—Mo1—K2	132.54 (9)	N6^viii^—K3—N7^ix^	88.30 (11)
C4—Mo1—K2	48.64 (10)	O3—K3—N7^ix^	69.5 (3)
C6—Mo1—K2	49.76 (10)	O1—K3—N7^ix^	119.81 (15)
C3—Mo1—K2	131.31 (10)	N1^ix^—K3—N7^ix^	87.22 (11)
C7—Mo1—K2	89.59 (10)	O2—K3—N1	76.9 (2)
C2—Mo1—K3	54.13 (11)	N6^viii^—K3—N1	151.78 (12)
C5—Mo1—K3	128.27 (11)	O3—K3—N1	118.3 (3)
C1—Mo1—K3	47.57 (9)	O1—K3—N1	133.85 (13)
C4—Mo1—K3	131.21 (10)	N1^ix^—K3—N1	75.93 (11)
C6—Mo1—K3	130.36 (11)	N7^ix^—K3—N1	80.28 (9)
C3—Mo1—K3	48.53 (10)	O2—K3—C1	83.5 (2)
C7—Mo1—K3	90.34 (10)	N6^viii^—K3—C1	154.61 (11)
K2—Mo1—K3	179.844 (19)	O3—K3—C1	124.7 (2)
N1^i^—K4—N2	74.36 (12)	O1—K3—C1	118.24 (13)
N1^i^—K4—N3^ii^	79.64 (10)	N1^ix^—K3—C1	79.58 (10)
N2—K4—N3^ii^	136.55 (11)	N7^ix^—K3—C1	102.07 (10)
N1^i^—K4—N3^iii^	155.68 (12)	N1—K3—C1	21.93 (10)
N2—K4—N3^iii^	128.12 (12)	O2—K3—C3	118.1 (2)
N3^ii^—K4—N3^iii^	76.83 (11)	N6^viii^—K3—C3	104.25 (11)
N1^i^—K4—N7^ii^	83.60 (11)	O3—K3—C3	129.1 (3)
N2—K4—N7^ii^	120.85 (12)	O1—K3—C3	80.92 (15)
N3^ii^—K4—N7^ii^	89.44 (11)	N1^ix^—K3—C3	78.63 (10)
N3^iii^—K4—N7^ii^	90.10 (11)	N7^ix^—K3—C3	158.45 (10)
N1^i^—K4—N4^iii^	88.28 (10)	N1—K3—C3	80.57 (10)
N2—K4—N4^iii^	70.89 (11)	C1—K3—C3	59.65 (10)
N3^ii^—K4—N4^iii^	74.16 (11)	O2—K3—N3	132.2 (2)
N3^iii^—K4—N4^iii^	91.13 (11)	N6^viii^—K3—N3	83.31 (10)
N7^ii^—K4—N4^iii^	162.80 (11)	O3—K3—N3	125.4 (3)
N1^i^—K4—N5	128.70 (12)	O1—K3—N3	74.17 (15)
N2—K4—N5	86.40 (12)	N1^ix^—K3—N3	74.35 (10)
N3^ii^—K4—N5	136.58 (11)	N7^ix^—K3—N3	160.97 (11)
N3^iii^—K4—N5	68.27 (11)	N1—K3—N3	99.33 (10)
N7^ii^—K4—N5	66.26 (12)	C1—K3—N3	79.57 (10)
N4^iii^—K4—N5	129.74 (12)	C3—K3—N3	20.96 (10)
N1^i^—K4—C3^iii^	155.52 (11)	O2—K3—C2	69.3 (2)
N2—K4—C3^iii^	111.36 (12)	N6^viii^—K3—C2	137.97 (11)
N3^ii^—K4—C3^iii^	80.82 (10)	O3—K3—C2	93.7 (2)
N3^iii^—K4—C3^iii^	20.96 (10)	O1—K3—C2	70.34 (13)
N7^ii^—K4—C3^iii^	110.84 (11)	N1^ix^—K3—C2	117.51 (11)
N4^iii^—K4—C3^iii^	72.25 (10)	N7^ix^—K3—C2	128.83 (11)
N5—K4—C3^iii^	75.78 (11)	N1—K3—C2	65.60 (11)
N1^i^—K4—C2	88.58 (11)	C1—K3—C2	47.92 (11)
N2—K4—C2	20.85 (11)	C3—K3—C2	48.92 (11)
N3^ii^—K4—C2	157.13 (11)	N3—K3—C2	66.16 (10)
N3^iii^—K4—C2	115.65 (11)	O2—K3—N2	54.2 (2)
N7^ii^—K4—C2	108.80 (11)	N6^viii^—K3—N2	130.22 (11)
N4^iii^—K4—C2	86.05 (11)	O3—K3—N2	73.6 (2)
N5—K4—C2	65.55 (11)	O1—K3—N2	56.52 (13)
C3^iii^—K4—C2	104.29 (11)	N1^ix^—K3—N2	137.62 (10)
N1^i^—K4—C4^iii^	109.02 (10)	N7^ix^—K3—N2	119.73 (11)
N2—K4—C4^iii^	77.45 (11)	N1—K3—N2	77.35 (10)
N3^ii^—K4—C4^iii^	78.98 (10)	C1—K3—N2	63.97 (10)
N3^iii^—K4—C4^iii^	72.38 (10)	C3—K3—N2	64.95 (10)
N7^ii^—K4—C4^iii^	160.72 (11)	N3—K3—N2	78.27 (10)
N4^iii^—K4—C4^iii^	20.75 (10)	C2—K3—N2	20.11 (10)
N5—K4—C4^iii^	112.42 (12)	C3—N3—K2^viii^	154.0 (3)
C3^iii^—K4—C4^iii^	52.41 (10)	C3—N3—K4^xi^	98.7 (3)
C2—K4—C4^iii^	86.57 (11)	K2^viii^—N3—K4^xi^	106.90 (12)
N1^i^—K4—C3^ii^	77.63 (10)	C3—N3—K4^iii^	89.6 (3)
N2—K4—C3^ii^	148.30 (11)	K2^viii^—N3—K4^iii^	89.18 (11)
N3^ii^—K4—C3^ii^	19.86 (10)	K4^xi^—N3—K4^iii^	103.17 (11)
N3^iii^—K4—C3^ii^	78.16 (9)	C3—N3—K3	78.8 (3)
N7^ii^—K4—C3^ii^	69.59 (10)	K2^viii^—N3—K3	92.09 (10)
N4^iii^—K4—C3^ii^	93.89 (10)	K4^xi^—N3—K3	99.76 (11)
N5—K4—C3^ii^	123.26 (11)	K4^iii^—N3—K3	155.59 (14)
C3^iii^—K4—C3^ii^	88.77 (10)	C4—N4—K1^iii^	151.2 (3)
C2—K4—C3^ii^	166.20 (10)	C4—N4—K2^vii^	99.5 (3)
C4^iii^—K4—C3^ii^	98.25 (10)	K1^iii^—N4—K2^vii^	109.07 (13)
N1^i^—K4—C7^ii^	80.29 (10)	C4—N4—K4^iii^	87.2 (3)
N2—K4—C7^ii^	136.66 (12)	K1^iii^—N4—K4^iii^	84.27 (10)
N3^ii^—K4—C7^ii^	69.42 (10)	K2^vii^—N4—K4^iii^	99.88 (11)
N3^iii^—K4—C7^ii^	85.77 (10)	C4—N4—K2	78.1 (3)
N7^ii^—K4—C7^ii^	20.02 (10)	K1^iii^—N4—K2	97.92 (11)
N4^iii^—K4—C7^ii^	143.19 (10)	K2^vii^—N4—K2	104.52 (11)
N5—K4—C7^ii^	82.72 (12)	K4^iii^—N4—K2	153.20 (14)
C3^iii^—K4—C7^ii^	106.31 (10)	C1—N1—K4^i^	154.3 (3)
C2—K4—C7^ii^	128.06 (11)	C1—N1—K3^ix^	99.3 (3)
C4^iii^—K4—C7^ii^	144.98 (11)	K4^i^—N1—K3^ix^	106.16 (12)
C3^ii^—K4—C7^ii^	49.57 (10)	C1—N1—K1^i^	86.9 (3)
N4^iii^—K1—O3	126.5 (3)	K4^i^—N1—K1^i^	84.65 (10)
N4^iii^—K1—N2	75.70 (12)	K3^ix^—N1—K1^i^	102.10 (11)
O3—K1—N2	79.8 (2)	C1—N1—K3	79.6 (3)
N4^iii^—K1—O2	132.7 (2)	K4^i^—N1—K3	97.05 (11)
O3—K1—O2	40.3 (3)	K3^ix^—N1—K3	104.07 (11)
N2—K1—O2	58.2 (2)	K1^i^—N1—K3	152.14 (14)
N4^iii^—K1—N7^iv^	83.94 (11)	C2—N2—K4	97.4 (3)
O3—K1—N7^iv^	72.6 (3)	C2—N2—K1	176.0 (4)
N2—K1—N7^iv^	124.93 (12)	K4—N2—K1	86.55 (12)
O2—K1—N7^iv^	112.8 (2)	C2—N2—K3	79.7 (3)
N4^iii^—K1—N6^i^	151.13 (12)	K4—N2—K3	176.65 (16)
O3—K1—N6^i^	73.8 (3)	K1—N2—K3	96.37 (12)
N2—K1—N6^i^	132.09 (12)	C5—N5—K2^vi^	176.1 (4)
O2—K1—N6^i^	76.2 (2)	C5—N5—K4	91.7 (3)
N7^iv^—K1—N6^i^	83.89 (11)	K2^vi^—N5—K4	85.04 (11)
N4^iii^—K1—N1^i^	90.63 (10)	C5—N5—K2	81.6 (3)
O3—K1—N1^i^	124.6 (3)	K2^vi^—N5—K2	101.74 (12)
N2—K1—N1^i^	71.04 (12)	K4—N5—K2	173.03 (16)
O2—K1—N1^i^	84.5 (2)	N6—C6—Mo1	178.8 (4)
N7^iv^—K1—N1^i^	160.62 (11)	N6—C6—K2	81.0 (3)
N6^i^—K1—N1^i^	92.27 (10)	Mo1—C6—K2	98.68 (14)
N4^iii^—K1—N6^iv^	79.25 (11)	N6—C6—K1^i^	71.9 (3)
O3—K1—N6^iv^	143.8 (2)	Mo1—C6—K1^i^	108.80 (14)
N2—K1—N6^iv^	135.46 (11)	K2—C6—K1^i^	146.00 (14)
O2—K1—N6^iv^	141.7 (2)	N6—C6—K1^xii^	62.6 (3)
N7^iv^—K1—N6^iv^	87.65 (11)	Mo1—C6—K1^xii^	116.23 (15)
N6^i^—K1—N6^iv^	74.18 (11)	K2—C6—K1^xii^	90.47 (10)
N1^i^—K1—N6^iv^	73.05 (10)	K1^i^—C6—K1^xii^	94.81 (10)
N4^iii^—K1—O1	77.78 (15)	N4—C4—Mo1	178.7 (4)
O3—K1—O1	48.9 (3)	N4—C4—K2	80.5 (3)
N2—K1—O1	59.89 (13)	Mo1—C4—K2	100.01 (13)
O2—K1—O1	70.8 (3)	N4—C4—K4^iii^	72.1 (3)
N7^iv^—K1—O1	66.03 (12)	Mo1—C4—K4^iii^	107.93 (13)
N6^i^—K1—O1	120.18 (14)	K2—C4—K4^iii^	144.67 (13)
N1^i^—K1—O1	130.92 (12)	N4—C4—K2^vii^	60.4 (3)
N6^iv^—K1—O1	146.53 (13)	Mo1—C4—K2^vii^	118.33 (15)
N4^iii^—K1—C6^i^	155.00 (11)	K2—C4—K2^vii^	96.36 (10)
O3—K1—C6^i^	78.4 (3)	K4^iii^—C4—K2^vii^	89.15 (10)
N2—K1—C6^i^	115.01 (12)	N5—C5—Mo1	174.1 (4)
O2—K1—C6^i^	66.0 (2)	N5—C5—K2	77.6 (3)
N7^iv^—K1—C6^i^	104.87 (11)	Mo1—C5—K2	96.91 (15)
N6^i^—K1—C6^i^	21.12 (10)	N5—C5—K4	68.2 (3)
N1^i^—K1—C6^i^	73.26 (10)	Mo1—C5—K4	117.19 (16)
N6^iv^—K1—C6^i^	77.82 (10)	K2—C5—K4	145.76 (14)
O1—K1—C6^i^	127.22 (14)	N1—C1—Mo1	179.0 (3)
N4^iii^—K1—C1^i^	112.02 (11)	N1—C1—K3	78.5 (3)
O3—K1—C1^i^	108.7 (3)	Mo1—C1—K3	101.55 (13)
N2—K1—C1^i^	78.95 (12)	N1—C1—K1^i^	71.7 (3)
O2—K1—C1^i^	71.3 (2)	Mo1—C1—K1^i^	108.74 (13)
N7^iv^—K1—C1^i^	154.97 (11)	K3—C1—K1^i^	141.57 (14)
N6^i^—K1—C1^i^	72.96 (10)	N1—C1—K3^ix^	60.7 (3)
N1^i^—K1—C1^i^	21.43 (10)	Mo1—C1—K3^ix^	118.36 (15)
N6^iv^—K1—C1^i^	77.16 (10)	K3—C1—K3^ix^	95.18 (10)
O1—K1—C1^i^	134.42 (12)	K1^i^—C1—K3^ix^	90.83 (9)
C6^i^—K1—C1^i^	52.82 (10)	N3—C3—Mo1	178.1 (4)
N4^iii^—K1—C7^iv^	78.81 (10)	N3—C3—K3	80.3 (3)
O3—K1—C7^iv^	90.8 (2)	Mo1—C3—K3	100.21 (13)
N2—K1—C7^iv^	139.24 (12)	N3—C3—K4^iii^	69.5 (3)
O2—K1—C7^iv^	129.9 (2)	Mo1—C3—K4^iii^	110.74 (14)
N7^iv^—K1—C7^iv^	19.95 (11)	K3—C3—K4^iii^	143.04 (14)
N6^i^—K1—C7^iv^	80.85 (10)	N3—C3—K4^xi^	61.5 (3)
N1^i^—K1—C7^iv^	140.67 (11)	Mo1—C3—K4^xi^	116.62 (15)
N6^iv^—K1—C7^iv^	67.80 (10)	K3—C3—K4^xi^	92.56 (10)
O1—K1—C7^iv^	84.04 (12)	K4^iii^—C3—K4^xi^	91.23 (10)
C6^i^—K1—C7^iv^	101.40 (10)	N2—C2—Mo1	175.1 (4)
C1^i^—K1—C7^iv^	140.79 (11)	N2—C2—K4	61.8 (3)
N3^v^—K2—N5^vi^	74.20 (12)	Mo1—C2—K4	123.11 (17)
N3^v^—K2—N4^vii^	79.00 (11)	N2—C2—K3	80.2 (3)
N5^vi^—K2—N4^vii^	143.65 (11)	Mo1—C2—K3	94.90 (14)
N3^v^—K2—N7^vii^	92.82 (10)	K4—C2—K3	141.94 (14)
N5^vi^—K2—N7^vii^	69.19 (12)	C6—N6—K3^v^	154.2 (3)
N4^vii^—K2—N7^vii^	88.33 (11)	C6—N6—K1^i^	87.0 (3)
N3^v^—K2—C4	154.84 (11)	K3^v^—N6—K1^i^	93.00 (11)
N5^vi^—K2—C4	130.86 (12)	C6—N6—K1^xii^	97.7 (3)
N4^vii^—K2—C4	78.98 (11)	K3^v^—N6—K1^xii^	107.02 (12)
N7^vii^—K2—C4	98.55 (10)	K1^i^—N6—K1^xii^	105.82 (11)
N3^v^—K2—N4	152.82 (11)	C6—N6—K2	78.0 (3)
N5^vi^—K2—N4	123.51 (12)	K3^v^—N6—K2	91.82 (10)
N4^vii^—K2—N4	75.48 (11)	K1^i^—N6—K2	154.44 (14)
N7^vii^—K2—N4	77.34 (10)	K1^xii^—N6—K2	96.69 (11)
C4—K2—N4	21.43 (10)	N7—C7—Mo1	178.9 (4)
N3^v^—K2—C6	103.06 (11)	N7—C7—K1^xii^	63.6 (2)
N5^vi^—K2—C6	131.04 (12)	Mo1—C7—K1^xii^	116.63 (15)
N4^vii^—K2—C6	78.58 (11)	N7—C7—K4^xi^	65.9 (3)
N7^vii^—K2—C6	156.93 (10)	Mo1—C7—K4^xi^	113.90 (14)
C4—K2—C6	60.55 (10)	K1^xii^—C7—K4^xi^	129.43 (15)
N4—K2—C6	81.01 (10)	N7—C7—K2^vii^	66.0 (3)
N3^v^—K2—N6	82.07 (9)	Mo1—C7—K2^vii^	115.03 (15)
N5^vi^—K2—N6	123.91 (12)	K1^xii^—C7—K2^vii^	83.05 (10)
N4^vii^—K2—N6	74.98 (11)	K4^xi^—C7—K2^vii^	74.38 (9)
N7^vii^—K2—N6	163.17 (11)	N7—C7—K3^ix^	62.5 (3)
C4—K2—N6	80.61 (10)	Mo1—C7—K3^ix^	116.39 (16)
N4—K2—N6	100.02 (10)	K1^xii^—C7—K3^ix^	77.65 (9)
C6—K2—N6	21.00 (10)	K4^xi^—C7—K3^ix^	82.09 (10)
N3^v^—K2—N5	127.59 (12)	K2^vii^—C7—K3^ix^	128.45 (14)
N5^vi^—K2—N5	78.26 (12)	C7—N7—K1^xii^	96.4 (3)
N4^vii^—K2—N5	138.08 (11)	C7—N7—K3^ix^	97.8 (3)
N7^vii^—K2—N5	117.52 (11)	K1^xii^—N7—K3^ix^	88.49 (10)
C4—K2—N5	65.63 (11)	C7—N7—K4^xi^	94.1 (3)
N4—K2—N5	78.79 (11)	K1^xii^—N7—K4^xi^	169.07 (15)
C6—K2—N5	65.05 (11)	K3^ix^—N7—K4^xi^	93.01 (11)
N6—K2—N5	77.64 (11)	C7—N7—K2^vii^	94.0 (3)
N3^v^—K2—C5	136.31 (12)	K1^xii^—N7—K2^vii^	93.66 (11)
N5^vi^—K2—C5	99.08 (12)	K3^ix^—N7—K2^vii^	167.66 (15)
N4^vii^—K2—C5	117.27 (11)	K4^xi^—N7—K2^vii^	82.64 (10)
N7^vii^—K2—C5	125.79 (11)	K3—O1—K1	102.27 (19)
C4—K2—C5	48.67 (11)	K3—O1—K2^viii^	85.71 (15)
N4—K2—C5	66.00 (11)	K1—O1—K2^viii^	169.2 (2)
C6—K2—C5	48.91 (11)	K3—O1—H1	133 (5)
N6—K2—C5	65.90 (11)	K1—O1—H1	109 (5)
N5—K2—C5	20.83 (10)	K2^viii^—O1—H1	70 (5)
N3^v^—K2—C4^vii^	79.86 (10)	K3—O1—H2	110 (4)
N5^vi^—K2—C4^vii^	128.22 (11)	K1—O1—H2	97 (5)
N4^vii^—K2—C4^vii^	20.13 (10)	K2^viii^—O1—H2	73 (5)
N7^vii^—K2—C4^vii^	68.23 (10)	H1—O1—H2	100 (3)
C4—K2—C4^vii^	83.64 (10)	K3—O2—K1	110.1 (4)
N4—K2—C4^vii^	72.96 (10)	O3^x^—O3—K3	124.8 (14)
C6—K2—C4^vii^	97.92 (10)	O3^x^—O3—K1	121.3 (14)
N6—K2—C4^vii^	95.04 (10)	K3—O3—K1	109.9 (3)
N5—K2—C4^vii^	149.11 (11)		

Symmetry codes: (i) −*x*, −*y*, −*z*+1; (ii) *x*+1, *y*, *z*; (iii) −*x*, −*y*, −*z*; (iv) *x*+1, *y*−1, *z*; (v) *x*, *y*+1, *z*; (vi) −*x*, −*y*+1, −*z*; (vii) −*x*−1, −*y*+1, −*z*; (viii) *x*, *y*−1, *z*; (ix) −*x*−1, −*y*, −*z*+1; (x) −*x*, −*y*−1, −*z*+1; (xi) *x*−1, *y*, *z*; (xii) *x*−1, *y*+1, *z*.
